# The Impact of COVID-19 on the Utilization of Public Sector Radiological Services in the Western Cape Province of South Africa

**DOI:** 10.7759/cureus.47616

**Published:** 2023-10-24

**Authors:** Nolene Teuteberg, Michelle M Barnard, Amanda Fernandez, Keith Cloete, Matodzi Mukosi, Richard Pitcher

**Affiliations:** 1 Division of Radiodiagnosis, Department of Medical Imaging and Clinical Oncology, Faculty of Medicine and Health Sciences, Stellenbosch University, Cape Town, ZAF; 2 Sub-Directorate Medical Imaging Services, Directorate: Health Technology, Western Cape Government Department of Health and Wellness, Cape Town, ZAF; 3 Department of Health and Wellness, Western Cape Government, Cape Town, ZAF

**Keywords:** public sector, healthcare, western cape province, south africa, middle-income country, utilisation, covid-19, trends, radiology

## Abstract

Background

Coronavirus (COVID-19) was officially declared a pandemic in March 2020 and has had a major impact on global healthcare services, including radiology. However, little is known about the full impact of COVID-19 on the utilization of diagnostic imaging in Africa’s public healthcare sector.

Objectives

The objective of this study was to compare public sector diagnostic imaging utilization by modality for the whole Western Cape Province (WCP) of South Africa (SA), as well as its metropolitan and rural areas, in 2019 and 2020 in terms of the absolute number of investigations and investigations per 1000 people.

Method

We performed a retrospective analysis of Western Cape Government Department of Health and Wellness and Stats SA District Council 2021 Mid-Year Population Estimates data. All diagnostic imaging investigations performed in 2019 and 2020 were collated and stratified by imaging modality, geographic region (metropolitan/rural), and calendar year. Data are presented as the total number of investigations and investigations per 1000^ ^people. We calculated mammography utilization for women aged 40-70 years and compared data for 2019 and 2020.

Results

Between 2019 and 2020, the provincial population increased by 1.9%, while total imaging investigations and investigations per 1000 people decreased by 19% (1,384,941 *vs.* 1,123,508, −261,433) and 20% (262/10^3^ *vs. *208/10^3^), respectively. Total numerical decline was highest in plain radiographs (1,005,545 *vs.* 800,641, −204,904), accounting for more than three-quarters (78%) of the total reduction.

Percentage decline was most pronounced for mammography, as utilization was almost halved (15.7/10^3^ *vs.* 8.9/10^3^, −43%), whereas computed tomography was the least impacted (17.9/10^3^ *vs.* 16.7/10^3^, −12%) with the remaining modalities decreasing between approximately one-quarter and one-fifth (magnetic resonance imaging = 26%, fluoroscopy = 25%, general radiographs = 23%, ultrasound = 16%, chest radiographs = 18%). Proportional metropolitan (−18.7%) and rural decreases (−19.3%) were similar.

Conclusion

COVID-19 had a substantial impact on WCP imaging services, decreasing overall radiological investigations by almost one-fifth. The greatest impact was on elective investigations, particularly mammography. Although the proportional impact was similar for the metropolitan and rural areas, COVID-19 nonetheless exacerbated existing discrepancies in imaging utilization between the geographical regions. The medium- and long-term clinical impacts of decreased imaging are still to be defined.

## Introduction

Severe acute respiratory syndrome coronavirus 2 was first reported in Wuhan, China, on December 31, 2019, and officially declared a pandemic on March 11, 2020. The first case in South Africa (SA) was documented on March 5, 2020, heralding the country’s first coronavirus disease 2019 (COVID-19) “wave,” which extended from March 2020 to November 2020 and peaked during the week of July 5-11 with 13,000 new cases daily. To curtail the spread of infection, a state of national disaster was declared on March 15 [1], and varying levels of restriction or lockdown were invoked for the remainder of 2020. During Level 5, the strictest lockdown (March 26-April 30), all citizens except essential workers were confined to their homes. Schools were closed, outdoor activities and public gatherings were prohibited, and all travel was suspended. Only essential goods were sold, with a complete ban on alcohol sales [2]. During Level 4 (May 1-31), mines, factories, and agriculture could resume limited operations with a phased return of employees. Exercise was permitted between 06:00 and 09:00 within a 5-km radius of home [3]. Public transport operated from 05:00 to 19:00 with restricted passenger numbers. Personal cars were restricted to three people per vehicle [2]. During Level 3 (June 1-August 17), schools reopened for grades 7-12 exercise was permitted from 06h00 to 18h00, and all retail shops were opened [2]. Interprovincial travel was still prohibited unless it was work-related and undertaken with the necessary permits. During Level 2 (August 18-September 20), interprovincial travel was permitted, and restaurants, bars, and taverns were opened to a maximum of 50 people, with onsite alcohol consumption permitted until 22:00 [4]. During Level 1 (September 21-December 28), international travel was allowed, and public venues were opened to 50% capacity [2]. Restrictions consistent with those of Levels 4 and 5 are broadly referred to as a “hard” lockdown.

The pandemic had a major impact on global healthcare, as national authorities, including SA, scaled down routine services and prioritized emergency responses. Outpatient attendance was curtailed, elective surgery was postponed, and staff redeployed from routine services to COVID wards and intensive care units [5-8]. Basic health services were disrupted in 90% of countries. Antenatal care, immunizations, family planning clinics, and the management of noncommunicable diseases were most affected, particularly in low- and middle-income countries [5]. In SA, the total number of visits to public healthcare facilities declined by 19%, from 99.6 million in 2019 to 81.2 million in 2020. At the provincial level, the Western Cape Province (WCP) and the Free State were the most and least impacted, recording declines of 31% and 9% in patient visits, respectively [8].

The pandemic also influenced the nature of emergency care. Individuals showed diminished health-seeking behavior, particularly for less severe illnesses, while trauma admissions declined. In New Zealand, the United Kingdom, France, and Spain, the overall trauma workload decreased by 37%-75% during hard lockdown [9-13]. SA reports from Grey’s Hospital, Pietermaritzburg, and Groote Schuur Hospital, Cape Town, showed 47% and 53% reductions in trauma workload, respectively, particularly involving motor vehicle accidents, pedestrian vehicle accidents, and assaults [14,15].

Given radiology’s integral role in healthcare [16] and its key function as a frontline COVID clinical service, the pandemic inevitably led to changes in diagnostic imaging [17]. Radiology departments experienced increased demand for emergency pulmonary imaging while rationalizing access to non-urgent and elective studies [18]. Additionally, in many instances, radiology staff were redeployed to clinical duties in COVID areas, which included internal medicine wards and COVID testing stations, and patients deferred routine investigations [18,19].

Hard lockdown data for April 2020 from Ohio, USA, documented a 55% overall reduction in imaging utilization compared to the previous year. Outpatient, emergency, and inpatient studies decreased by 68%, 48%, and 31%, respectively, while mammography, ultrasound (US), magnetic resonance imaging (MRI), plain radiography, and computed tomography (CT) decreased by 93%, 58%, 56%, 53%, and 47%, respectively. Central and peripheral hospital utilization declined by 42% and 54%-64%, respectively [20]. Single-institution data from Pakistan, comparing imaging workload in 2019 and 2020, showed decreases in all primary imaging modalities during the pandemic, with US, plain radiographs, MR, and CT showing 37%, 30%, 14%, and 2% reductions, respectively [17]. Similar data during the first COVID wave in Milan, Italy, documented reduced workloads for all modalities, body regions, and types of care, except CT, which increased by 3% [18]. England’s National Health Service (NHS) recorded a 22% overall decrease in diagnostic imaging utilization between 2019-2020 and 2020-2021, with substantially higher reductions in screening mammography (44%) than those in plain radiography (27%), fluoroscopy (26%), MR (21%), US (20%), and CT (7%) [21-23].

Studies of the impact of COVID-19 on imaging utilization have been limited to North America, Europe, and Asia [16,17,19]. There has been no such assessment in Africa. Such a study is particularly important in SA, given the country’s unique quadruple burden of disease, with high levels of communicable and non-communicable disease, maternal/child mortality, and violence [24,25]. Data from countries with different disease profiles and healthcare infrastructure cannot be extrapolated for the environment of SA. An understanding of the impact of the COVID pandemic on imaging services in SA is useful for informing strategic planning for future COVID waves or pandemics, and it would also provide key insights into any potential imaging backlog to be addressed. SA’s WCP is the ideal setting for such a study. A detailed description of WCP public-sector imaging infrastructure and utilization was recently published for the decade preceding the COVID pandemic (i.e., 2009-2019), which showed that population growth was the main driver of imaging utilization and that the province had an average annual increment of 1.4 imaging studies per thousand people [26]. The aim of this study was to analyze changes in WCP public-sector imaging utilization between 2019 and 2020.

## Materials and methods

This retrospective audit was conducted in the WCP, the southernmost of SA’s nine provinces, with approximately 12% of the national population [1,27]. The WCP is divided into six administrative districts. The Cape Town Metropolitan District, with more than 60% of the provincial population but just 2% of the land area, is surrounded by five sprawling rural districts [26,28]. 

WCP health services are based on mirrored, tiered referral pathways for rural and metropolitan areas [26]. Initial imaging access is typically at community centers, with subsequent referral to district, regional, and central hospitals with progressive access to more specialized imaging modalities [29]. The central hospitals are university-affiliated tertiary-level teaching institutions.

A digital imaging platform is used across the WCP, which has an amalgamated picture-archiving and communication system across various levels of care. Most facilities can view imaging studies performed across the platform, eliminating unnecessary service duplication. The Medical Imaging Services Sub-Directorate (MISSD) within the Directorate of Health Technology (DOHT) in the Western Cape Government Department of Health and Wellness is tasked with the collation of all data pertaining to the utilization of provincial diagnostic imaging services. These data include the utilization of services at each facility and form the basis of this study.

We extracted imaging utilization data for 2019 and 2020 from the MISSD database and stratified them by imaging modality (i.e., plain radiography, US, fixed and mobile fluoroscopy, CT, MRI, and whole-body digital radiography) and geographical location (i.e., rural/metropolitan). Population statistics for 2019 and 2020 were based on Stats SA District Council 2021 Mid-Year Population Estimates, acquired using a cohort component methodology, and released at mid-year based on the latest available information [1]. We calculated the number of imaging studies performed per 1000 people reliant on the public healthcare sector (75% of the WCP population) [30] by modality for 2019 and 2020 for the whole province, as well as for the metropolitan and rural areas. We compared the ratio of metropolitan to rural studies performed across modalities for 2019 and 2020, excluding MRI, low-dose X-ray, and digital subtraction angiography, which represent shared resources.

We stratified plain radiographs as either chest X-rays (CXR) or general X-rays. The workload was analyzed by the absolute number of investigations and investigations per 1000 people for each modality, for the whole province, as well as the metropolitan and rural areas. We used breast imaging analyses per 1000 women aged 40-70 years in keeping with a previous WCP analysis of radiological utilization [26].

The study was approved by the Health Research Ethics Committee (HREC) of the Faculty of Medicine and Health Sciences at Stellenbosch University and by the Health Research Committee of the WCP under the auspices of the National Health Research Database (HREC Reference No. S21/11/020_COVID-19; Project Reference No. 24205).

## Results

Provincial analysis

Population

In the review period, the WCP population grew by 1.86% (n = 127,861; 6,886,690 vs. 7,014,551), as seen in Table 1.

**Table 1:** Total metropolitan, rural, and provincial imaging studies for 2019 and 2020. Source: Stats Sa and MISSD [1]. *Public health care % for WCP is 75.9% [30]. Note: CT = computed tomography, MRI = magnetic resonance imaging, DSA = digital subtraction angiography, LODOX = low-dose X-ray.

	METROPOLITAN						RURAL						WESTERN CAPE					
	2019		2020		Total increase (n)	% increase	2019		2020		Total increase (n)	% increase	2019		2020		Total increase (n)	% increase
	n	%	n	%			n	%	n	%			n	%	n	%		
Total population	4517676	0	4607601	0	89925	1.99	2369014	0	2406950	0	37936	1.6	6886690	0	7014551	0	127861	1.86
Population reliant on public healthcare*	3428916.084	0	3497169.159	0	68253.075	1.99	1798081.626	0	1826875.05	0	28793.424	1.6	5226997.71	0	5324044.209	0	97046	1.86
Female population (40-70)	691316	0	711091	0	19775	2.86	350456	0	357184	0	6727.533496	1.9	1041772	0	1068275	0	26503	2.54
Female population (40-70) reliant on public healthcare	524709	0	539718.069	0	15009	2.86	265996	0	271103	0	5106.552	1.9	790705	0	810821	0	20116	2.54
Chest X-Ray	333624	35.5	276455	36.2	-57169	-17.1	137658	33.6	111251	33.6	-26407	-19.2	471282	34	387706	34.5	-83576	-17.7
General X-Ray	351127	37.4	269719	35.4	-81408	-23.2	183136	44.7	143216	43.3	-39920	-21.8	534263	38.6	412935	36.8	-121328	-22.7
Total X-Ray	684751	72.9	546174	71.6	-138577	-20.2	320794	78.3	254467	76.9	-66327	-20.7	1005545	72.6	800641	71.3	-204904	-20.4
Ultrasound	147389	15.7	126518	16.6	-20871	-14.2	70961	17.3	61204	18.5	-9757	-13.7	218350	15.8	187722	16.7	-30628	-14
CT	79255	8.4	71286	9.3	-7969	-10.1	14164	3.5	12485	3.8	-1679	-11.9	93419	6.7	83771	7.5	-9648	-10.3
Mammography	10772	1.1	5970	0.8	-4802	-44.6	1644	0.4	1225	0.4	-419	-25.5	12416	0.9	7195	0.6	-5221	-42.1
Fluoroscopy	16644	1.8	12932	1.7	-3712	-22.3	2396	0.6	1651	0.5	-745	-31.1	19040	1.4	14583	1.3	-4457	-23.4
MRI	0	0	0	0	0	0	0	0	0	0	0	0	12641	0.9	9470	0.8	-3171	-25.1
DSA	0	0	0	0	0	0	0	0	0	0	0	0	17441	1.3	15312	1.4	-2129	-12.2
LODOX	0	0	0	0	0	0	0	0	0	0	0	0	6089	0.4	4814	0.4	-1275	-20.9
TOTAL studies	938811	100	762880	100	-175931	-18.7	409959	100	331032	100	-78927	-19.3	1384941	100	1123508	100	-261433	-18.9

Overall Imaging Utilization

Overall provincial imaging workload decreased by 19%, or more than a quarter of a million studies (n = 261,433; 1,384,941 vs. 1,123,508). In absolute numbers, the decline was most striking for plain radiographs, which decreased by more than 200,000 investigations (n = 201,604) and accounted for more than three-quarters (78%) of the total reduction. 

The most striking percentage decrease was in mammography, where utilization was almost halved (12,416 vs. 7195; −42%), representing a total decline of 5221 studies. CT was proportionally the least impacted modality, reduced by only 10%, which nonetheless equated to almost 10,000 fewer studies (n = 9648; 93,419 vs. 83,771). Other modalities decreased between approximately one-fifth and one-quarter (MRI = 25%, fluoroscopy = 23%, general radiographs = 23%, US = 14%, chest radiographs = 18%). The proportion of basic radiological investigations (plain radiographs and ultrasound combined) remained relatively constant, being 88.4% and 88.0% in 2019 and 2020, respectively.

Imaging Utilization Per 1000 People

Overall investigations per 1000 people decreased by one-fifth, or 54 studies (265 vs. 211). Proportional decreases by modality were broadly aligned with those for absolute numbers, as seen in Table 2.

**Table 2:** Metropolitan, rural, and provincial studies per 1000 people for 2019 and 2020. Source: Stats SA and MISSD. Note: CT = computed tomography, MRI = magnetic resonance imaging, DSA = digital subtraction angiography, LODOX = low-dose X-ray.

	METROPOLITAN				RURAL				WESTERN CAPE			
	2019	2020	Total increase (n/1000 people)	Total % increase per 1000 people	2019	2020	Total increase (n/1000 people)	Total % increase per 1000 people	2019	2020	Total increase (n/1000 people)	% increase
Total population	4517676	4607601	89925	1.99	2369014	2406950	37936	1.60	6886690	7014551	127861	1.86
Population reliant on PHC*	3428916.1	3497169.2	68253.1	1.99	1798081.6	1826875.1	28793.4	1.60	5226997.7	5324044.2	97046	1.86
Female population (40-70)	691316	711091	19775	2.86	350456	357184	6728	1.92	1041772	1068275	26503	2.54
Female population (40-70) reliant on PHC	524709	539718	15009	2.86	265996	271103	5107	1.92	790705	810821	20116	2.54
Chest X-Ray	97.3	79.1	-18.2	-18.8	76.6	60.9	-15.7	-20.5	90.2	72.8	-17.3	-19.2
General X-Ray	102.4	77.1	-25.3	-24.7	101.9	78.4	-23.5	-23.0	102.2	77.6	-24.7	-24.1
Total X-Ray	199.7	156.2	-43.5	-21.8	178.4	139.3	-39.1	-21.9	192.4	150.4	-42.0	-21.8
Ultrasound	43.0	36.2	-6.8	-15.8	39.5	33.5	-6.0	-15.1	41.8	35.3	-6.5	-15.6
CT	23.1	20.4	-2.7	-11.8	7.9	6.8	-1.0	-13.2	17.9	15.7	-2.1	-12.0
Mammogram	20.5	11.1	-9.5	-46.1	6.2	4.5	-1.7	-26.9	15.7	8.9	-6.8	-43.5
Fluoroscopy	4.9	3.7	-1.2	-23.8	1.3	0.9	-0.4	-32.2	3.6	2.7	-0.9	-24.8
MRI	0	0	0	0	0	0	0	0	2.4	1.8	-0.6	-26.5
DSA	0	0	0	0	0	0	0	0	3.3	2.9	-0.5	-13.8
LODOX	0	0	0	0	0	0	0	0	1.2	0.9	-0.3	-22.4
TOTAL studies	273.8	218.1	-55.7	-20.3	228	181.2	-46.8	-20.5	265	211	-53.9	-20.4

Analysis comparing metropolitan and rural areas

Population

Although there was an expansion of both the metropolitan (4,517,676 vs. 4,607,601; 1.99%) and rural (2,369,014 vs. 2,406,950; 1.60%) populations, proportional metropolitan growth exceeded rural growth by 24% (1.99% vs. 1.60%). 

Overall Imaging Utilization

Combined utilization of plain radiography, US, fluoroscopy, CT, and mammography decreased by 18.7% (938,811 vs. 762,880) and 19.3% (409,959 vs. 331,032) in the metropolitan and rural areas, respectively, with similar proportional reductions in the rural and metropolitan areas (19.3% vs. 18.8%, respectively). 

Imaging Utilization Per 1000 People

In 2019, combined metropolitan utilization of plain radiography, US, fluoroscopy, mammography, and CT (274 studies/10^3^ people) exceeded rural utilization (228 studies/10^3^ people) by approximately 46 studies/10^3^ people (20%). The most striking discrepancies by modality were for mammography (20.5 vs. 6.2, 231%), CT (23.1 vs. 7.9, 192%), fluoroscopy (4.9 vs. 1.3, 277%), and CXR (97.3 vs. 76.6, 27%). General radiography (102.4 vs. 101.9) and US (43.0 vs. 39.5) utilization were similar in the two areas. 

In 2020, both metropolitan and rural regions recorded decreased combined utilization of the above-mentioned modalities (i.e., plain radiography, US, fluoroscopy, mammography, and CT) per 1000 people. However, the proportional rural decrease (228 vs. 181, 21%) was slightly greater than the metropolitan decrease (274 vs. 218, 20%), with the resultant increased discrepancy between metropolitan and rural utilization (nine studies/10^3^ people, 20%). However, the patterns of impact were different across the modalities for the two regions. The proportional reduction in metropolitan mammography utilization (20.5 vs. 11.1, 46%) exceeded that of rural areas (6.2 vs. 4.5, 27%), whereas the proportional rural reduction in fluoroscopy utilization (1.3 vs. 0.9, 32%) was higher than that of metropolitan areas (4.9 vs. 3.7, 24%).

## Discussion

To the best of our knowledge, this is the only assessment of the impact of the COVID-19 pandemic on public sector imaging in Africa. It is also the most comprehensive analysis of the pandemic’s influence on radiological services in a low- or middle-income country. As such, it has the potential to enhance understanding of the effects of the pandemic on the continent and beyond. 

There were several key findings in our study. The first was the sheer scale of the impact of COVID-19 on a single province in one year, decreasing overall radiological investigations by more than a quarter of a million, or 19% of all studies. The second was the wide differential impact by modality, with mammography showing the greatest proportional reduction (42%), being approximately four times that of CT (10%) and almost double that of the remaining modalities. The third was the relatively small difference in proportional impact, with the exception of mammography, between the metropolitan and rural areas. This, nonetheless, exacerbated existing discrepancies in imaging utilization between metropolitan and rural areas. The fourth was the broad alignment of findings from this study with those of similar assessments in other healthcare settings.

The WCP data show close congruence with those of NHS England [22], which recorded a 22% year-on-year decrease in investigations, equating to 9.9 million studies, with plain radiographs (n = 6.33 million) accounting for approximately two-thirds (64%) of the total reduction. Of note, NHS reductions were greatest for elective activity, being referrals from outpatient or general practitioner appointments. Referrals from emergency departments and in-patients were less impacted, with some modalities, notably plain radiographs and CT, contributing to COVID diagnostics [22].

The extent of the impact of COVID-19 on elective WCP investigations is exemplified by the 42% decrease in mammography studies for 2020. The clinical impact of such a contracted mammography service is reflected in the 44% reduction (247 vs. 139) in new breast cancers diagnosed at a large WCP anatomical pathology laboratory in the first three months of lockdown (1 April to 30 June 2020), compared to the corresponding period in 2019 [31]. Substantial decreases in mammographic investigations are common in all reports on the impact of COVID-19 on radiological departments [32-34]. The American Mammography Database recorded a 42% reduction in diagnostic studies during peak COVID lockdown, compared to pre-COVID levels, with a corresponding 52% reduction in breast cancer diagnoses. Furthermore, post-COVID breast cancer diagnoses did not reach those in the pre-COVID era, contributing to a growing cumulative diagnostic deficit from the start of the pandemic [35]. An American study showed that cancers diagnosed during COVID were of slightly increased size and node positivity [36]. There were similar findings in Brazil, where a 49% reduction in breast cancer diagnoses was recorded in the first wave of the pandemic. Detected cases had a worse prognosis, tending to be symptomatic women with palpable masses and more aggressive tumor subtypes. Indolent tumors proved more vulnerable to mammography service interruptions [37]. The NHS recorded a 44% reduction in screening mammography between 2019 and 2020, with a corresponding 39% reduction in breast cancer detection and an 8% increase (8.4 vs. 9.1) in cancers detected per 1000 women screened [23].

It is widely acknowledged that the full clinical significance of curtailed COVID-19 mammographic services is yet to be revealed globally [38]. In the WCP, the most immediate and quantifiable impact is the deficit of approximately 5221 investigations for the year 2020 alone, with the majority (n = 4802, 92%) being in the metropolitan area. Addressing the metropolitan backlog will be a major challenge, given that the pre-COVID metropolitan mammography service was deemed to be at full capacity. It is noteworthy that this study highlighted the known discrepancy [39] in mammography utilization between metropolitan and rural areas. The finding that the proportional decrease in rural mammography investigations (25%) was less than that of metropolitan investigations (45%) is likely attributable to the low baseline of rural mammographic utilization (6.2 studies/10^3^ eligible women) compared to the metropolitan baseline (20.5 studies/10^3 ^eligible women). 

One strength of this study is its foundation on accurate data from all WCP public sector diagnostic imaging facilities, correlated with provincial population data over a two-year period. As such, it provides a robust overview of the impact of the pandemic on a provincial imaging system. The reported data are unique in the SA context and, to our knowledge, across low- and middle-income countries. The work allows appreciation of the elective imaging backlog in certain modalities in the metropolitan and rural areas of the WCP and can assist in future resource allocation, including potential redeployment of staff and resources in the post-COVID era, to address backlogs, particularly in mammography.

The study is limited by its retrospective design and the absence of comprehensive demographic data for the imaged population. Additionally, the clinical indications for and acuity of investigations are not provided, while ultrasound, CT, and MRI studies were not stratified by body part. These shortcomings preclude detailed analyses of the clinical impact of decreased imaging during the pandemic. These limitations are common to other published work on the impact of COVID on radiological services and underscore the need for ongoing analyses of the medium- and long-term clinical impacts of the pandemic.

## Conclusions

COVID-19 had a substantial impact on WCP imaging services, decreasing overall radiological investigations by almost one-fifth. The greatest impact was on elective investigations, particularly mammography. Although the proportional impact was similar for the metropolitan and rural areas, COVID-19 nonetheless exacerbated existing discrepancies in imaging utilization between the geographical regions. The medium- and long-term clinical impacts of decreased imaging are still to be defined.
